# Should fraction flow reserve be considered an important decision-making tool to stratify patients with stable coronary artery disease for percutaneous coronary intervention? A meta-analysis

**DOI:** 10.1097/MD.0000000000008748

**Published:** 2017-11-17

**Authors:** Pravesh Kumar Bundhun, Chakshu Gupta, Feng Huang

**Affiliations:** a Institute of Cardiovascular Diseases, the First Affiliated Hospital of Guangxi Medical University; b Guangxi Medical University; c Institute of Cardiovascular Diseases and Guangxi Key Laboratory Base of Precision Medicine in Cardio-cerebrovascular Diseases Control and Prevention, the First Affiliated Hospital of Guangxi Medical University, Nanning, Guangxi, P. R. China.

**Keywords:** Fraction flow reserve, major adverse cardiac events, percutaneous coronary intervention

## Abstract

**Background:**

Nowadays, fraction flow reserve (FFR) is being discussed in every percutaneous coronary intervention (PCI) capable hospitals. Owing to recent development in the medical field, FFR-guided PCI should be able to find a place in Interventional Cardiology. At present, the importance of FFR to stratify patients who require PCI has seldom systematically been investigated. In this analysis, we aimed to compare the major adverse cardiac events (MACEs) mainly in patients with stable coronary artery disease (CAD) to whom PCI was recommended and deferred respectively based on the FFR value.

**Methods:**

Electronic databases were searched for studies comparing FFR-recommended versus FFR-deferred coronary stenting. Long-term MACEs, mortality, and myocardial infarction (MI) were considered as the clinical endpoints in this analysis. Odds ratios (ORs) with 95% confidence intervals (CIs) were calculated and the analyses were carried out by the latest version of the RevMan software.

**Results:**

A total number of 1753 patients (670 patients were revascularized, whereas 1083 patients were deferred from revascularization based on the FFR value) were analyzed. Current results showed MACEs and MI were significantly higher in the FFR-recommended PCI group with OR 1.34 (95% CI: 1.05–1.72; *P* = .02) and OR 1.73 (95% CI: 1.19–2.51; *P* = .004, *I*^2^ = 0%), respectively. However, mortality was similarly manifested with OR 1.23 (95% CI: 0.92–1.63; *P* = .16, *I*^2^ = 0%).

**Conclusion:**

Significantly higher MACEs were observed in patients to whom PCI was recommended compared to those patients who were deferred from undergoing PCI based on the FFR values. Therefore, FFR might indeed be an important decision-making procedural tool, which should be used to stratify stable CAD patients with an advanced disease and who are qualified candidates for PCI. Further research should confirm this hypothesis.

## 1 Introduction

Nowadays, fraction flow reserve (FFR) is being discussed in every percutaneous coronary intervention (PCI) capable hospitals. Owing to recent development in the medical field, FFR guided PCI should be able to find a place in Interventional Cardiology.

In coronary artery diseases (CAD), FFR can provide a functional evaluation or assessment of the obstructed artery.^[1]^ With reference to the repeated noninvasive stress testing, the FFR threshold to differentiate or to recognize a clinically significant lesion level ischemia is 0.75.^[2]^ Nevertheless, to expand measurement sensitivity to exclude the presence of functionally significant stenosis, a threshold of 0.80 has recently been adopted by the European Society of Cardiology (ESC).^[3]^

However, when a decision is to be taken with the involvement of FFR, other clinical factors should also be taken into consideration. An FFR value lying between 0.76 and 0.80 was considered to be in the “grey zone.” Even if clinical practice guideline has adopted a cutoff value of ≤0.80, several previous studies have considered FFR value of ≤0.75 as the cutoff value.

At present, the importance of FFR to stratify patients who need to be revascularized with PCI has seldom systematically been investigated. In this analysis, we aimed to compare the major adverse cardiac events (MACEs) mainly in patients with stable CAD to whom PCI was recommended or deferred based on the FFR value.

## 2 Methods

### 2.1 Data sources and search strategy

Studies comparing FFR-recommended versus FFR-deferred coronary stenting were searched from electronic databases (MEDLINE: database of medical articles, EMBASE, and the Cochrane library) by typing the terms “fraction flow reserve and percutaneous coronary intervention.” The abbreviations “FFR and PCI” as well as the terms “FFR-deferred angiography, deferred” were also used. In addition, reference lists of selective articles were also checked for relevant studies. This search strategy was only based on articles, which were published in English. Publications which were available in Chinese or other non-English languages were not considered relevant.

### 2.2 Inclusion and exclusion criteria

Studies were included if:

1. They were randomized trials or observational studies comparing FFR-recommended versus FFR-deferred PCI.
2. They had a follow-up period of >1 year.
3. MACEs, myocardial infarction (MI), or mortality were reported among their clinical endpoints.

Studies were excluded if they were meta-analyses, case studies, and letter to editors even if they were associated with FFR-guided PCI.

1. They only compared FFR-guided with non-FFR-guided angiography or PCI.
2. They had a short-term follow-up period of <1 year.
3. They did not report MACEs, MI, or mortality among their clinical endpoints.
4. They were duplicated studies.

### 2.3 Type of participants, outcomes, definitions, and follow-up

Those who participated in this analysis comprised of patients with stable CAD, intermediate, or moderate coronary stenosis as shown in Table 1.

**Table 1:**
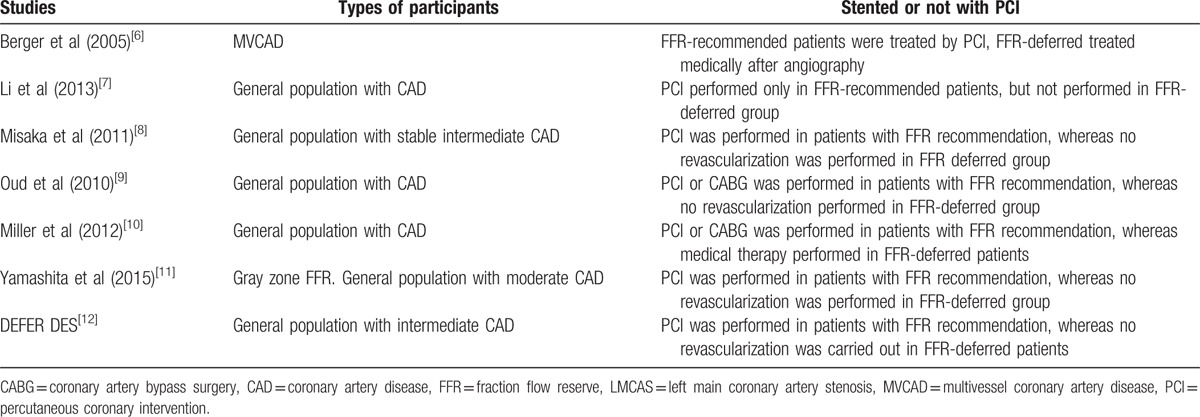
Types of participants.

The outcomes which were analyzed included:

1. MACEs (mortality, MI, and repeated revascularization)
2. Mortality (all-cause death/cardiac death)
3. MI

This analysis had a long-term follow-up period of >1 year.

The clinical outcomes which were reported in each study, as well as the corresponding follow-up periods, were listed in Table 2.

**Table 2:**
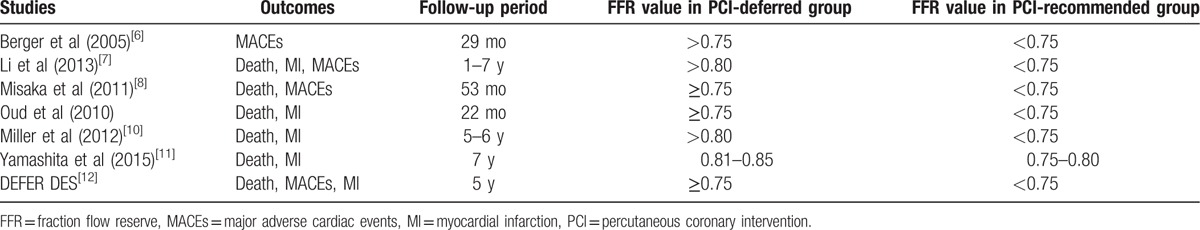
Reported outcomes and follow-up periods.

### 2.4 Data extraction and review

The first (PKB) and the second authors (CG) individually assessed the titles and abstracts which were presented during the search strategy, and selected the full-text articles, which were qualified for this analysis. The type of study which was reported, the total number of patients in the FFR-recommended PCI group, and the FFR-deferred group, respectively, patients’ characteristics at baseline, and the outcomes which were reported with respective follow-up periods were systematically extracted and cross-checked carefully. Any disagreement about including certain studies or data was resolved by the third author (FH). The PRISMA (Preferred Reporting Items for Systematic Reviews and Meta-Analyses)^[4]^ reporting guideline was relevant to this research.

### 2.5 Statistical analysis

Heterogeneity was assessed using 2 specific statistical tests, which were frequently being used in meta-analyses^[5]^ namely: the Cochrane Q-statistic test and the *I*^2^ statistic test. For the Cochrane *Q* statistic test, statistical significance was shown by a *P* value ≤0.05. Concerning the *I*^2^ statistical test, if the percentage of *I*^2^ was low, a low heterogeneity was assumed (fixed-effects model was used), whereas if the percentage of *I*^2^ was high, we assumed a high level of heterogeneity (a random-effects model was used). Ethical or board review approval was not applicable for this study.

Sensitivity analysis was also carried out by an exclusion method. In addition, funnel plots were used to visually estimate publication bias (other methods were not appropriate because of a very small number of studies which was included). Odds ratios (OR) with 95% confidence intervals (CIs) were generated and the analysis was carried out by the latest version of the RevMan software (RevMan 5.3).

## 3 Results

### 3.1 Searched outcomes

**Figure 1:**
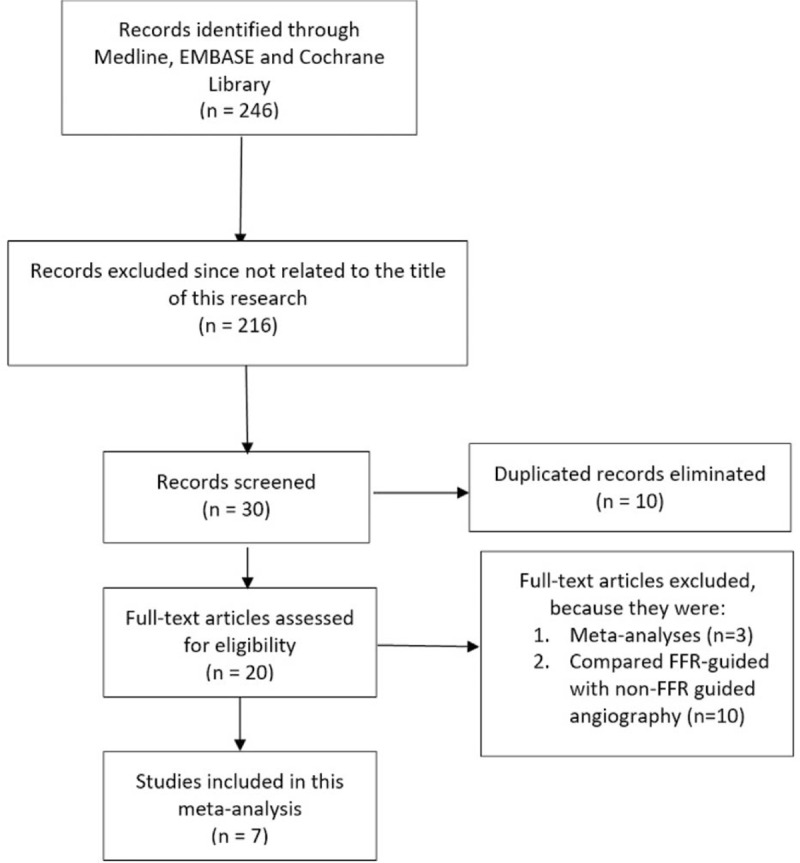
Flow diagram representing the study selection. FFR = fraction flow reserve.

Two hundred and forty-six (246) articles were obtained from electronic databases. After a careful assessment and review of the titles and abstracts, 216 articles which were not related to the idea of this analysis were directly eliminated. In addition, another 10 articles were eliminated as they were duplicates of the studies which were initially considered relevant. Twenty full-text articles were assessed for eligibility. Ten articles were further eliminated as they compared FFR-guided with non-FFR-guided coronary angiography. Another 3 articles were eliminated as they were meta-analyses. Finally, 7 articles^[6–12]^ were confirmed for this meta-analysis (Fig. 1).

### 3.2 General features of the studies

A total number of 1753 patients (670 patients were revascularized, whereas 1083 patients were deferred from revascularization based on the FFR value) were analyzed. Table 3 summarized the main features of the studies which were included in this analysis.

**Table 3:**
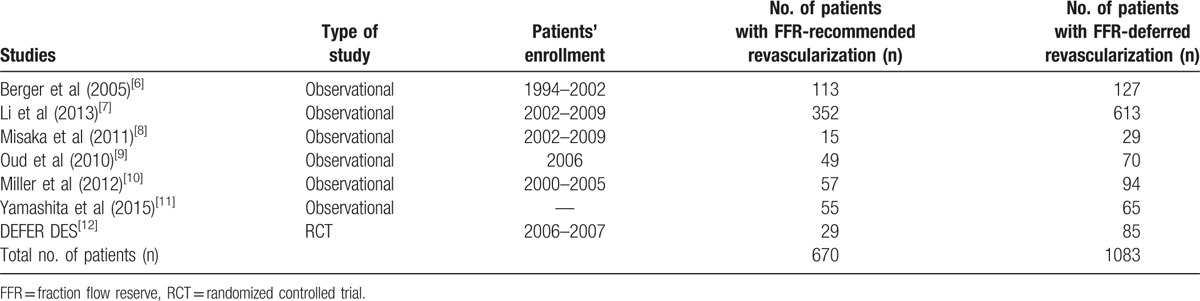
General features of the studies which were included.

### 3.3 Baseline features of the patients

The baseline features of the patients have been summarized in Table 4.

**Table 4:**
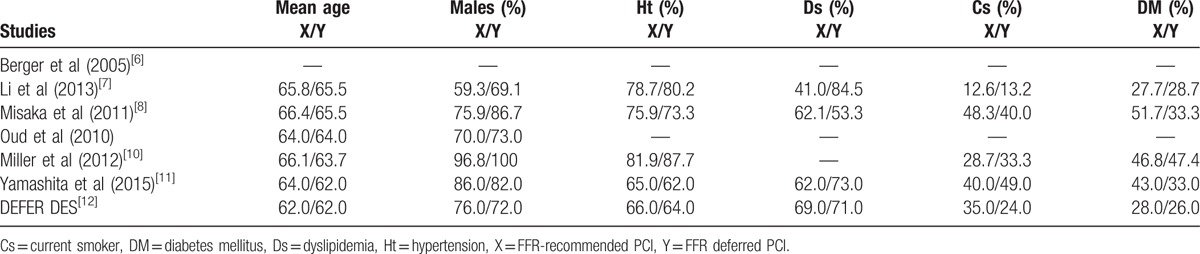
Baseline features of the studies which were included.

Mean age was reported in years. According to Table 4, no significant differences were observed in baseline features (except for dyslipidemia which was lower in the FFR-deferred group) among patients who were classified in the FFR-recommended PCI group and patients to whom PCI was deferred.

### 3.4 Major adverse cardiac events which were reported in patients to whom PCI was recommended versus patients to whom PCI was deferred based on the FFR value

The results of this analysis have been represented in Table 5.

**Table 5:**
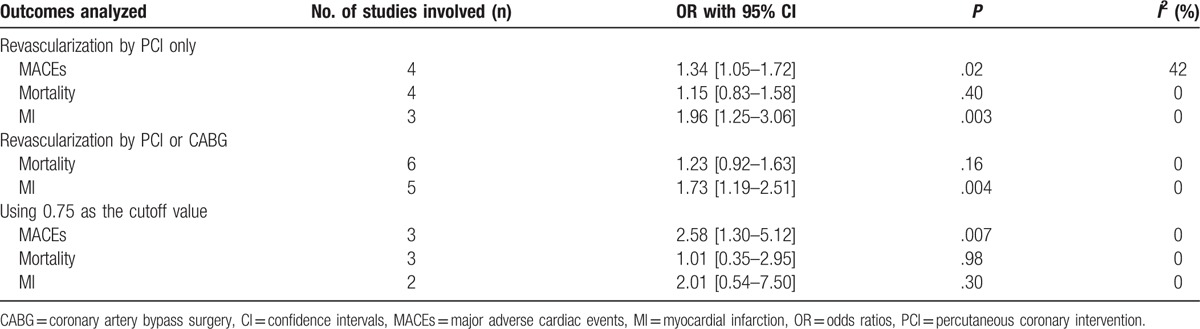
Results of this analysis.

In this analysis, a few studies also reported patients who were revascularized by coronary artery bypass surgery (CABG) or PCI based on the FFR value. Because the total number of patients who underwent revascularization with PCI or CABG were combined in 2 studies,^[9,10]^ and the respective data could not be separated, this analysis was carried out twice (once without those 2 studies and another time including both studies).

**Figure 2:**
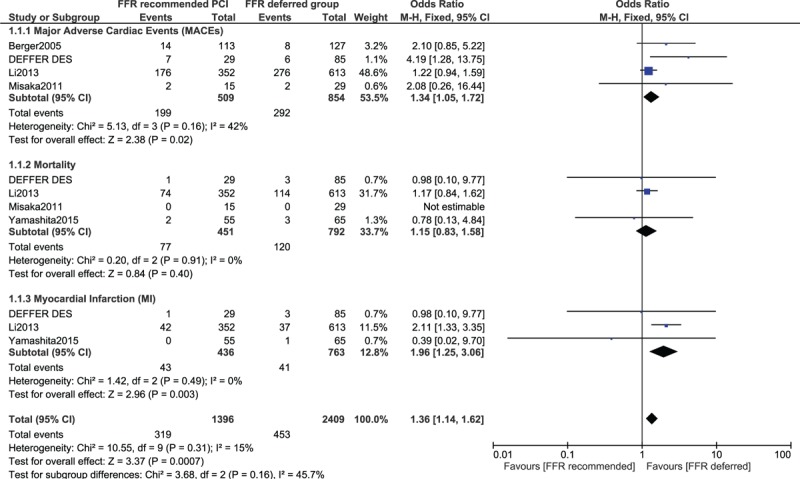
Adverse cardiovascular outcomes observed between FFR-recommended versus FFR-deferred coronary intervention (including patients who were revascularized only by PCI). CI = confidence interval, FFR = fraction flow reserve, PCI = percutaneous coronary intervention.

MACEs and MI were significantly higher in those patients who underwent PCI with OR 1.34 (95% CI: 1.05–1.72; *P* = .02) and OR 1.96 (95% CI: 1.25–3.06; *P* = .003), respectively compared to those patients to whom PCI was deferred based on the FFR value. However, mortality was not significantly different with OR 1.15 (95% CI: 0.83–1.58; *P* = .40) as shown in Figure 2.

**Figure 3:**
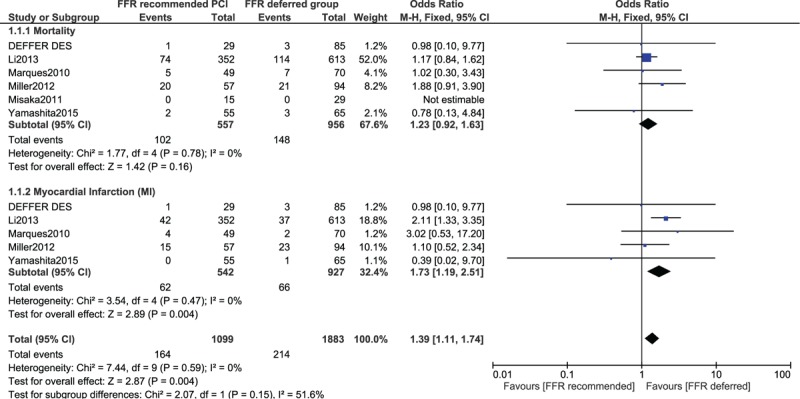
Adverse cardiovascular outcomes observed between FFR-recommended versus FFR-deferred coronary intervention (including patients who were revascularized by PCI or coronary artery bypass surgery). CI = confidence interval, FFR = fraction flow reserve, PCI = percutaneous coronary intervention.

When studies of Oud et al (2010)^[9]^ and Miller et al (2012)^[10]^ were included from the main analysis, MI was still significantly higher in patients to whom PCI/CABG was recommended with OR 1.73 (95% CI 1.19–2.51; *P* = .004), whereas no significant difference was observed in mortality among patients to whom revascularization was recommended or deferred based on the FFR value with OR 1.23 (95% CI: 0.92–1.63; *P* = .16) as shown in Figure 3.

As this analysis included different cut-off values of FFR, whereby in some cases, PCI was deferred with an FFR value >0.75 or >0.80, and recommended in patients with an FFR value <0.75 or <0.80, we additionally carried out a selective analysis including only studies, which reported an FFR value of 0.75 as the cutoff point.

**Figure 4:**
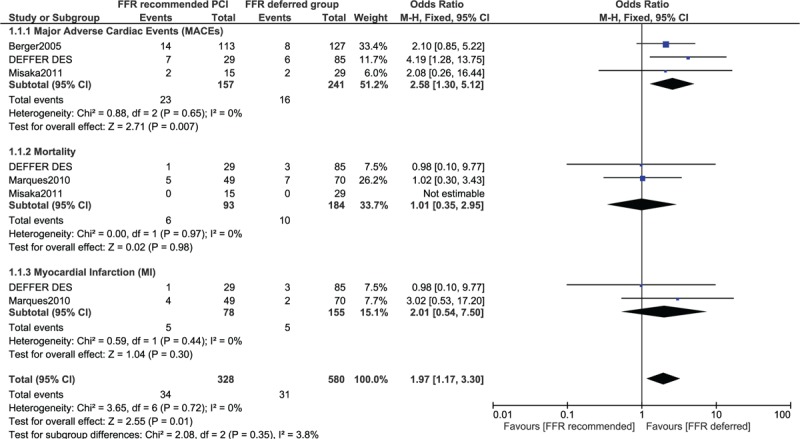
Adverse cardiovascular outcomes observed between FFR-recommended versus FFR-deferred coronary intervention (PCI or CABG using an FFR value 0.75 as the cutoff point). CI = confidence interval, FFR = fraction flow reserve, PCI = percutaneous coronary intervention.

This selective analysis also showed MACEs to be significantly higher in the FFR-recommended revascularization group with OR 2.58 (95% CI: 1.30–5.12; *P* = .007). Mortality and MI were not significantly different with OR 1.01 (95% CI: 0.35–2.95; *P* = .98) and OR 2.01 (95% CI: 0.54–7.50; *P* = .30), respectively as shown in Figure 4.

**Figure 5:**
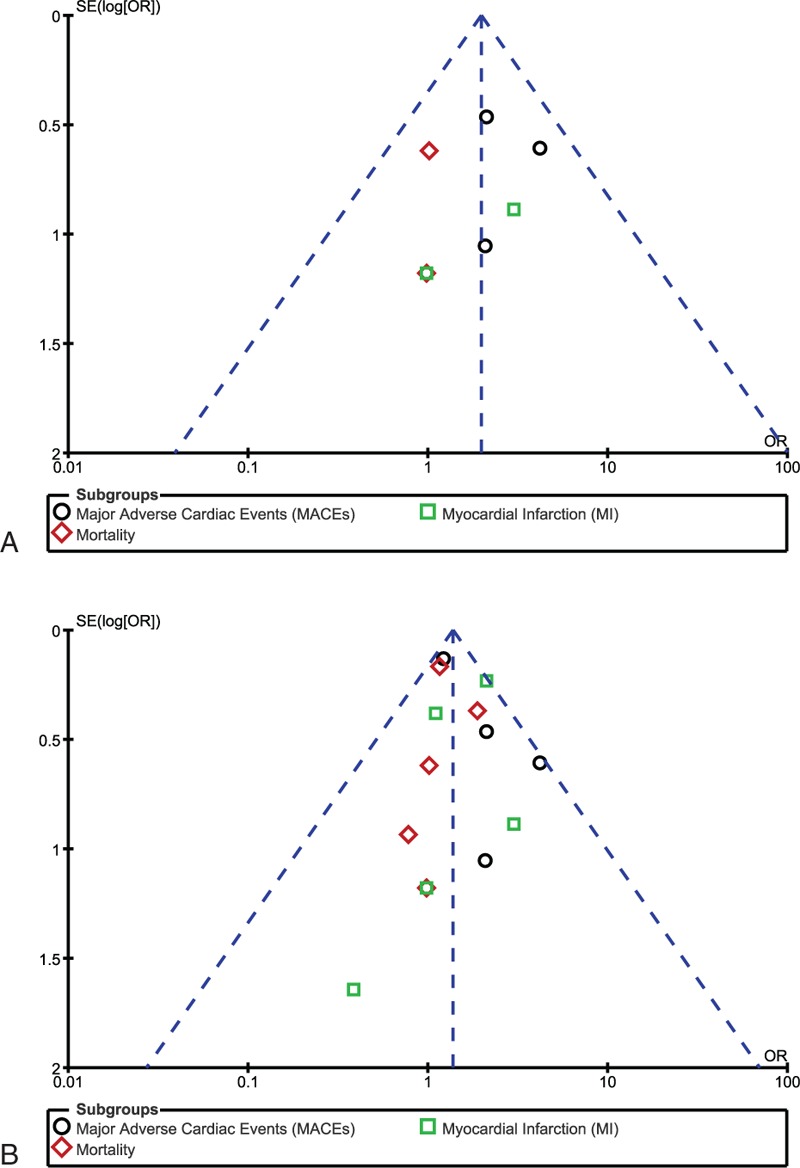
(A and B) Funnel plots showing publication bias.

Sensitivity analysis yielded consistent results. Moreover, there was only little evidence of publication bias based on visually assessing the funnel plots (Fig. 5A and B).

## 4 Discussion

This is the first systematic review and meta-analysis comparing the long-term MACEs based on patients to whom PCI was recommended or deferred based on the FFR value. Several studies have already shown that FFR-guided angiography is safe and will be beneficial in PCI. However, this current analysis further showed that in all the patients to whom FFR-guided angiography was considered, FFR-recommended PCI indicated an advanced CAD, which could later be associated with a higher risk of MACEs compared to patients to whom PCI was deferred. This hypothesis should be considered a new feature of this analysis. Moreover, being associated with a very low level of heterogeneity among all the subgroups which were analyzed, this study represents another new feature.

Results of this analysis showed a significantly higher rate of MACEs and MI in patients to whom revascularization was recommended based on the FFR value compared to patients to whom revascularization was deferred.

Kim et al^[13]^ showed that FFR-guided PCI was very safe and effective among the 131 patients with multiple intermediate stenosis. Moreover, their study did not show any event related to deferred lesions. The authors also concluded that FFR-guided PCI could decrease unnecessary interventions and maximize the benefits of PCI with drug-eluting stents.

Results of the DEFER (FFR to Determine Appropriateness of Angioplasty in Moderate Coronary Stenoses) study^[14]^ showed that every 0.01 decrease in FFR value was associated with a significantly higher rate of death because of cardiac cause and a higher rate of MI in patients with acute coronary syndrome (among 1872 patients who underwent FFR assessment between the years 2002 and 2012).

However, the current analysis showed a significantly higher rate of MACEs and MI in the FFR-recommended PCI group, whereas the result for mortality was not significant.

Pereira et al^[15]^ assessed the long-term follow-up of patients with deferred coronary intervention guided by measurement of FFR. They showed MACEs-free survival to be 97.8% during a follow-up period of 1 year among the 232 patients who were involved. In their study, patients had intermediate CAD (50%–70% of coronary obstruction) and they were deferred for coronary intervention based on an FFR value <0.80 as adopted by the ESC.

In addition, Dominguez-Franco et al^[16]^ assessed the long-term prognosis in diabetic patients to whom revascularization was deferred following FFR assessment. PCI was deferred in 136 patients with an FFR value ≥0.75 and in their study, the authors concluded that deferring PCI in diabetic patients with moderately severe coronary artery obstruction having an FFR of ≥0.75 was considered safe and was not associated with long-term MACEs. However, other research showed diabetes mellitus to be independently associated with target vessel failure when compared to nondiabetic patients with deferred FFR.^[17]^

This current analysis showed that significantly higher major adverse events were observed in patients to whom PCI was recommended compared to patients to whom PCI was deferred based on the FFR value showing that the latter could be considered as an important decision-making tool to predict whether a coronary disease is severe enough to consider revascularization by PCI, thus selecting those patients with mild CAD who are at low risk for MACEs and who do not require revascularization.

However, further research should focus on the disadvantage of FFR, if present.

Recently, Bundhun et al^[18]^ showed FFR-guided PCI not to be associated with significantly increased adverse outcomes when compared to angiography-guided PCI. Inspired by their research, we carried out this analysis which was based on patients to whom PCI was recommended or deferred based on the FFR value.

This current analysis consisted of data which were obtained mainly from observation studies (except DEFER DES trial) because there was only 1 randomized trial that satisfied the inclusion and exclusion criteria of this study. However, the best part of this analysis (positive aspect) was the involvement of a very low level of heterogeneity among all the subgroups which were analyzed.

Even if this analysis represents a completely new idea in clinical medicine, it also has several limitations. First of all, because of the limited number of patients that were analyzed, this study might not provide robust results. In addition, study of Berger et al (2005) did not indicate the number of patients in the FFR-recommended and FFR-deferred groups; therefore, when carrying out the statistical analysis, the number of lesions were included instead. Moreover, even if all the studies which were involved reported a long-term follow-up period, the period interval was different in each study. This might also have affected the results of this analysis. The inclusion of data from observational cohorts could also represent a limitation. In addition, the revascularization procedures, the severity of CAD, and the postinterventional medications could all have had an impact on the results. At last, even if this analysis was based mainly on patients with stable CAD, 1 study which was also included in this analysis consisted of patients with multivessel CAD.

## 5 Conclusion

Significantly higher MACEs were observed in patients to whom PCI was recommended compared to those patients who were deferred from undergoing PCI based on the FFR values. Therefore, FFR might indeed be an important decision-making procedural tool which should be used to stratify stable CAD patients with an advanced disease and who are qualified candidates for PCI. Further research should confirm this hypothesis.
